# Myeloma Overexpressed 2 (Myeov2) Regulates L11 Subnuclear Localization through Nedd8 Modification

**DOI:** 10.1371/journal.pone.0065285

**Published:** 2013-06-12

**Authors:** Manato Ebina, Fuminori Tsuruta, Megumi C. Katoh, Yu Kigoshi, Akie Someya, Tomoki Chiba

**Affiliations:** Graduate School of Life and Environmental Sciences, University of Tsukuba, Tsukuba, Ibaraki, Japan; University of Pittsburgh School of Medicine, United States of America

## Abstract

Nucleolus is a dynamic structure that controls biogenesis of ribosomal RNA and senses cellular stresses. Nucleolus contains a number of proteins including ribosomal proteins that conduct cellular stresses to downstream signaling such as p53 pathway. Recently, it has been reported that modification by a ubiquitin-like molecule, Nedd8, regulates subnuclear localization of ribosomal protein L11. Most of L11 is normally localized and neddylated in nucleolus. However, cellular stress triggers deneddylation and redistribution of L11, and subsequent activation of p53. Although Nedd8 modification is thought to be important for L11 localization, the mechanism of how neddylation of L11 is regulated remains largely unknown. Here, we show that Myeloma overexpressed 2 (Myeov2) controls L11 localization through down-regulation of Nedd8 modification. Expression of Myeov2 reduced neddylation of proteins including L11. We also found that Myeov2 associates with L11 and withholds L11 in nucleoplasm. Although Myeov2 interacted with a Nedd8 deconjugation enzyme COP9 signalosome, L11 deneddylation was mediated by another deneddylase Nedp1, independently of Myeov2. Finally, p53 transcriptional activity is upregulated by Myeov2 expression. These data demonstrate that Myeov2 hampers L11 neddylation through their interactions and confines L11 to nucleoplasm to modulate nucleolar integrity. Our findings provide a novel link between oncogenic stress and p53 pathway and may shed light on the protective mechanism against cancer.

## Introduction

Nucleolus is a non-membrane organelle composed of ribosomal DNA and proteins. The major functions of nucleolus are synthesis of ribosomal RNA (rRNA) and ribosomal subunit assembly. In addition, nucleolus acts as a sensor of cellular stress response and transmits signals to stress response pathways [1], [2]. Exposure to a variety of stresses such as DNA damage, heat shock, and inhibition of DNA and RNA synthesis causes nucleolus disruption, leading to activation of p53, which is the primary mediator of the stress response [1], [2]. Recent studies have identified a number of nucleolar proteins such as ribosomal proteins, INK4a/ARF (ARF), PICT1 and Nucleophosmin (NPM) [3], [4], [5], those are crucial for the regulation of stress-induced activation of p53 pathway. For instance, subsets of ribosomal proteins are implicated in stress-induced p53 activation and one of well-characterized proteins is L11 [6], [7], [8], [9], [10], [11], [12], [13]. L11 is localized in nucleolus under normal condition and supports nuclear integrity. On the other hand, L11 is released to nucleoplasm upon nucleolar stress, and binds to and inhibits Mdm2, the major p53 ubiquitin ligase, leading to p53 activation [6], [7] or recruitment of co-activator p300/CBP to p53 promoter site [14]. Thus, L11 is one of the key mediators that link nucleolar stress to p53 pathway. In addition, ARF, a tumor suppressor protein, is normally localized in nucleolus through interaction with NPM [15]. However, in response to nucleolar stress, ARF redistributes to nucleoplasm and interacts with p53 ubiquitin ligase, Mdm2, followed by suppression of Mdm2 activity [4], [16], [17], [18], [19]. Consequently, p53 accumulates in nucleus and induces target gene expression. Furthermore, it has recently been reported that another nucleolar protein, PICT1 interacts with L11, and confines L11 to nucleolus. However, PICT1 releases L11 into nucleoplasm following nucleolar stress, leading to suppression of Mdm2 [5]. Taken together, nucleolus acts as a central hub for tuning cellular stress response, and nucleolar stress alters properties of proteins in nucleolus and conducts signaling to p53 pathway.

It has been demonstrated that several nucleolar stress-related proteins including L11 are modified by a ubiquitin-like protein, Nedd8. A number of ribosomal proteins are usually neddylated in vivo [20]. This neddylation plays an important role in regulating the stability and subcellular localization of these proteins. In response to nucleolar stress, L11 is deneddylated by Nedd8 deconjugation enzyme, Nedp1/Den1/SENP8, and translocates from nucleolus to nucleoplasm, leading to an induction of p53 activity [21]. On the other hand, Mdm2 has also been reported to be a target of Nedd8 modification by itself [22]. Mdm2 possesses an E3 ligase activity of not only ubiquitin but also Nedd8. Mdm2 is autoneddylated to prevent degradation in resting state and is capable of both ubiquitination and neddylation of p53. However, treatment with chemotherapeutic agent such as doxorubicin promotes Nedd8 deconjugation from Mdm2, destabilizes and degrades Mdm2, followed by accumulation of p53 [23]. Thus, these observations suggest that Nedd8 pathway is crucial for the regulation of stress response. However despite the importance of Nedd8 pathway, the mechanisms by which stresses control Nedd8 modification are not fully understood.

Multiple myeloma is a plasma cell cancer. Plasma cells differentiate from B cells in bone marrow, and produce a huge number of specific antibodies [24], [25]. Plasma cells have unique “wheel-spoke” like chromatin structure and their nucleolus cannot be normally observed. However, once plasma cells transform into plasmacytoma, enlarged nucleoli appear in nucleus [26], [27]. Therefore, abnormal nucleolus is considered to be a pathological feature in multiple myeloma. So far, it has been reported that several proteins are associated with multiple myeloma [24], [25]. Among them, Myeloma overexpressed 2 (Myeov2) was initially reported as a 57 amino acid protein [28], but was later discovered to be a 252 amino acid protein (www.ensembl.org). Myeov2 is evolutionarily conserved from plant to human, and thought to have similar functions among different species. Given that Myeov2 is expressed abnormally in myeloma cells, expression level should be tightly regulated to maintain normal state and it is likely that Myeov2 expression is associated with the onset of multiple myeloma. However, it is largely unknown how Meyov2 is regulated.

In this study, we found that Myeov2 regulates L11 subnucleolar localization and consequently Nedd8 modification. Overexpression of Myeov2 led to a decrease of neddylated L11 and high proportion of nucleoplasmic L11. Intriguingly, Myeov2 interacted with COP9 signalosome, a Nedd8 deconjugation enzyme, although the interaction was not essential for Myeov2-mediated L11 deneddylation. Because expression level of Myeov2 is associated with multiple myeloma, our findings suggest that Myeov2 is a primary mediator that regulates nucleolus integrity and stress response pathway.

## Results

### Myeov2 Associates with COP9 Signalosome

**Figure 1:**
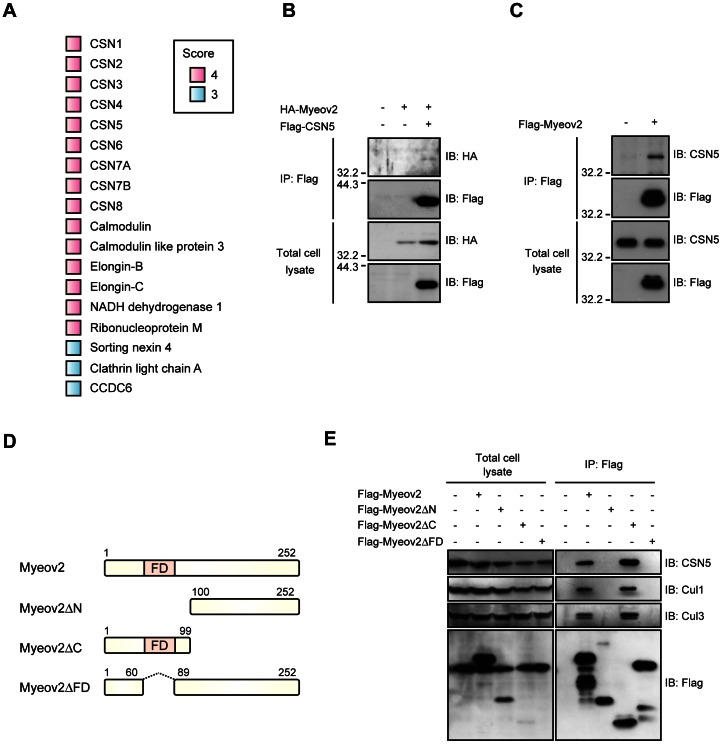
Myeov2 associates with COP9 signalosome. (**A**) Protein identified by mass spectrometry of Myeov2 immunoprecipitants. The colored squares show how often a protein was identified in multiple experiments. (**B**) Co-immunoprecipitation of HA-Myeov2 and Flag-CSN5 in HEK293T cells. Cell lysates were immunoprecipitated with anti-Flag antibody and analyzed using Flag and HA antibodies. (**C**) Co-immunoprecipitation of Flag-Myeov2 and endogenous CSN5 in HEK293T cells. Cell lysates were immunoprecipitated with anti-Flag antibody and analyzed using Flag and CSN5 antibodies. (**D**) Schematic structure of Myeov2 deletion mutants. (**E**) Co-immunoprecipitation of CSN5, Cul1, and Cul3 with Flag-Myeov2 in HEK293 cells. Cell lysates were immunoprecipitated with anti-Flag antibody and analyzed using indicated antibodies.

Myeov2 is overexpressed in myeloma cells and appears to be associated with the plasmacytes proliferation and transformation. Although expression level of Myeov2 is considered to be important for the tumorigenesis of plasma cells, little is known about the function of Myeov2. To investigate this, we searched for target proteins that interact with Myeov2 in HEK293 cells. We identified 18 proteins that associate with Myeov2 at least three times of four experiments (score≥3). Surprisingly, all subunits of COP9 signalosome were co-precipitated with Flag-Myeov2 (Fig. 1A). COP9 signalosome is a protein complex composed of 8 subunits (CSN1 to CSN8) that possesses deneddylation activity toward Cullin family proteins, which are components of Cullin-RING ubiquitin ligase (CRLs) [29], [30]. Given that deconjugating activity of COP9 signalosome is crucial for CRLs-dependent cellular proliferation and viability, we hypothesized that COP9 signalosome and CRLs are functional targets of Myeov2. To verify those interactions by immunoprecipitation experiment, we co-transfected HEK293T cells with HA-Myeov2 and Flag-CSN5, a catalytic subunit of COP9 signalosome. Immunoprecipitation of Flag-CSN5 resulted in co-immunoprecipitation of HA-Myeov2 (Fig. 1B). Reciprocal immnoprecipitation experiment by transfecting the cells with Flag-Myeov2, revealed the association of Flag-Myeov2 with endogenous CSN5, indicating that Myeov2 associates with CSN5 (Fig. 1C). To further explore the domain important for the interaction, we made deletion mutants of Myeov2 and introduced these constructs into HEK293T cells (Fig. 1D). We found that a FD region, which is a phenylalanine-aspartic acid rich domain and well conserved among different species, is essential for the binding with CSN5. Furthermore, FD region was also important for the association with Cullin family proteins (Fig. 1E). Taken together, these data imply that COP9 signalosome is a putative target of Myeov2.

### Expression of Myeov2 maintains L11 in a Lower Neddylation Level

**Figure 2:**
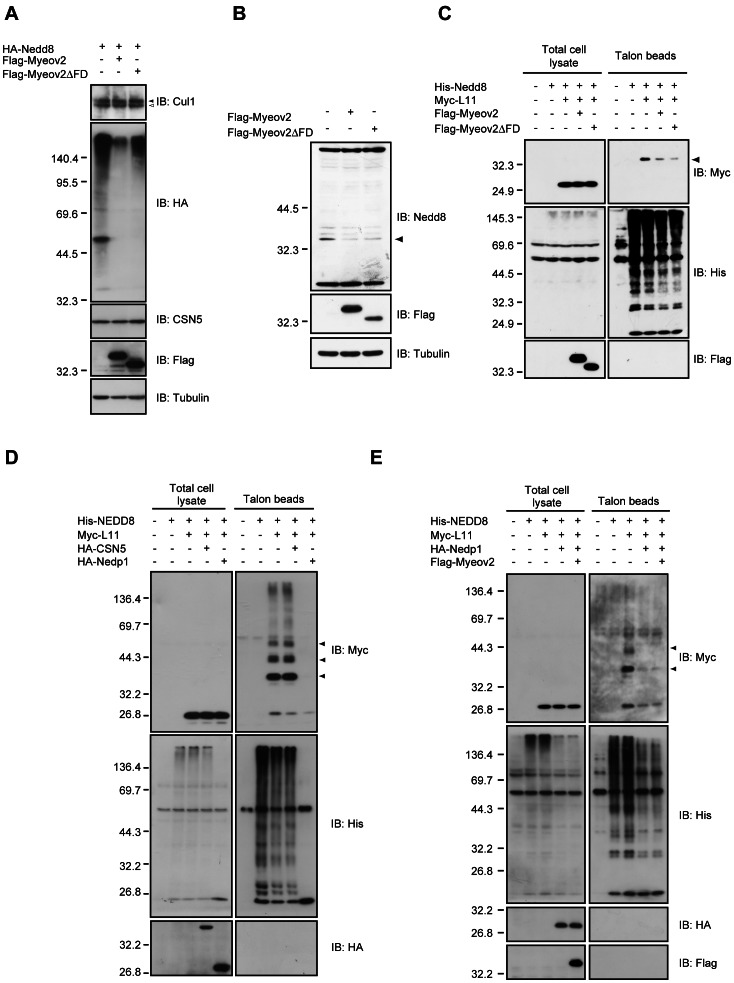
Expression of Myeov2 maintains L11 in a lower neddylation level in cells. (**A**) HEK293 cells were transfected with HA-Nedd8 and either Flag-Myeov2 or Flag-Myeov2ΔFD. Cell lysates were subjected to immunoblot analysis using indicated antibodies. (**B**) HEK293T cells were transfected with either Flag-Myeov2 or Flag- Myeov2ΔFD and then treated with 10 µM MG132 for 1 hour prior to harvest. Cell lysates were subjected to immunoblot analyses. Arrowhead indicates putative Myeov2 target protein. (**C, D and E**) HEK293T cells were transfected as indicated. Neddylated proteins were precipitated from denatured cell lysates using Talon metal affinity resin and immunoblotted with indicated antibodies. Arrowheads indicate neddylated L11.

COP9 signalosome mediates the deneddylation of Cullin family proteins and regulate CRLs activity; however, the mechanisms of how COP9 signalosome is activated are not well understood. Because our data imply that Myeov2 could be another component of COP9 signalosome, we asked whether Myeov2 mediates deneddylating activity. Unexpectedly, expression of Flag-Myeov2 did not change the neddylation status of Cul1, a cullin family protein (Fig. 2A). Thus, these data suggest that Myeov2 does not enhance deneddylation activity of COP9 signalosome at least in this system. Meanwhile, we found that expression of Myeov2 led to a global decrease of neddylated protein under the same experimental condition (Fig. 2A), and dominant negative CSN5 does not suppress this effect (data not shown). In addition, Myeov2ΔFD, which cannot interact with COP9 signalosome, also reduced the proportion of neddylated protein. However, as neddylation status was examined by ectopic expression of HA-Nedd8, it was possible that variability in transfection efficiency among each sample have affected the proportion of neddylated proteins. To avoid this possibility, we detected endogenous neddylated proteins using anti-Nedd8 antiserum (See Fig.S1 for the characterization of antiserum). The Nedd8 antiserum recognized a number of endogenous neddylated proteins (Fig. S1). When film exposure time was shortened, bands of around 30∼40 kDa proteins were still detected even though most bands became undetectable (Fig. 2B). Interestingly, the expression of both Myeov2 and Myeov2ΔFD significantly decreased these bands, especially a band of ∼35 kDa protein (Fig. 2B). Therefore, we speculated that these proteins, especially ∼35 kDa protein, are potent target(s) of Myeov2. Several proteins including a subset of ribosomal proteins have been reported to be neddylated so far and the molecular weights of those ribosomal proteins are around 15∼30 kDa [20]. Among several ribosomal proteins that are neddylated, we focused on L11, whose molecular weight is estimated to be 35kDa upon neddylation. L11 is modified by Nedd8 at multiple sites, and plays pivotal roles in regulation of p53 pathway. Thus we hypothesized that ∼35 kDa protein is L11 conjugated by monomeric or multimeric Nedd8. To examine this, we transfected cells with His-Nedd8 and Myc-L11, and precipitated neddylated proteins using Talon metal affinity beads under denatured condition. As expected, precipitated Myc-L11 was covalently conjugated with His-Nedd8 and expression of Myeov2 reduced the amount of precipitated L11. In addition, Myeov2ΔFD, which cannot associate with COP9 signalosome, also reduced the amount of precipitated L11 similar to Myeov2 (Fig. 2C).

We next investigated the molecular mechanisms of how expression of Myeov2 causes a decrease of neddylation status of L11. To explore this question, we focused on another deneddylating enzyme, Nedp1, which possesses a deneddylation activity for a broad range of proteins in cells [31]. We transfected cells with His-Nedd8, HA-Nedp1 and HA-CSN5 as indicated. When His-Nedd8 was precipitated using Talon metal affinity beads, multiple neddylated proteins were co-precipitated. Co-expression of Nedp1 clearly reduced this multiple bands, while that of CSN5 had no effect, suggesting that Nedp1, but not COP9 signalosome, is a primary deneddylating enzyme for L11 (Fig. 2D). We next investigated whether Myeov2 enhances Nedp1 activity. Because Nedp1 has a strong enzymatic activity in our system (Fig. 2D), we used lower amount of Nedp1 construct to detect the effect of Nedp1 activity in the presence or absence of Myeov2. Co-expression of Myeov2 did not further enhance Nedp1 activity (Fig. 2E), suggesting that Myeov2 has little effect on the regulation of Nedp1 activity. We also examined whether Myeov2 is necessary for the reduction of L11 neddylation level. To do this, we developed short hairpin RNAs that target Myeov2 (shMyeov2). Knockdown of Myeov2 has little effect on neddylation level of L11, implying that Myeov2 is not necessary for the inhibition of L11 neddylation (Fig.S2). Finally, Myeov2 did not interact with Nedp1 (Fig. S3), supporting our data that Myeov2 is not involved in Nedp1-mediated L11 deneddylation, and this may propose a potential mechanism by which Myeov2 leads to a decrease of neddylated L11 through suppression of neddylation and contributes to maintain a small proportion of neddylated L11 in cells.

### Myeov2 Alters L11 Subnuclear Localization without Nucleolar Disruption

**Figure 3:**
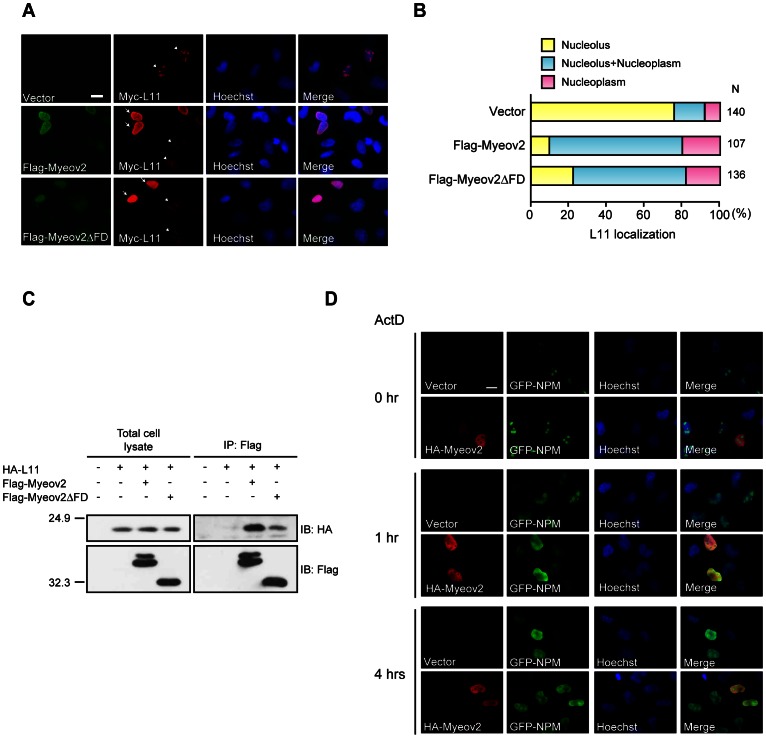
Myeov2 alters L11 subnuclear localization without nucleolar disruption. (**A**) HeLa cells were transfected with Myc-L11, together with either Flag-Myeov2 or Flag- Myeov2ΔFD plasmids, and subjected to immunocytochemistry. Arrows and arrowheads indicate Myeov2-expressing and control cells, respectively. Scale bar, 20 µm. (**B**) Quantification of the data in (A). Subnuclear localization of Myc-L11 in Flag-Myeov2-positive cells was examined. Cells that express L11 in nucleous only (yellow), both in nucleolus and nucleoplasm (blue) and nucleoplasm only (pink), were counted. N denotes the number of cells counted. (**C**) Co-immunoprecipitation of Flag-Myeov2 or Flag-Myeov2ΔFD with HA-L11 in HEK293T cells. Cell lysates were immunprecipiated with anti-Flag antibodies and analyzed using both Flag and HA antibodies. (**D**) HeLa cells transfected with EGFP-NPM and HA-Myeov2 were treated with 10 nM ActD for indicated time, and subjected to immunocytochemistry. Expression of Myeov2 did not induce nucleolar disruption in the absence of ActD (top panels). Nuclear disruption was observed in Myeov2-expressing cells at 1 hr of ActD treatment (middle panels). 4 hrs of ActD treatment resulted in nucleolar disruption irrespective of Myeov2 expression (lower panels). Scale bar, 20 µm.

L11 is usually localized in nucleolus and is relocated to nucleoplasm in response to nucleolar stress, followed by activation of p53 pathway. In addition, Nedd8 pathway is thought to be important for subnuclear localization of L11 [21]. We thus examined if Myeov2 regulates L11 subnuclear localization. Most of L11 were localized in nucleolus; however, expression of Myeov2 increased a population of nucleoplasmic L11. Myeov2ΔFD also confined L11 in nucleoplasm, although its effect was milder compared with full length Myeov2 (Fig. 3A and 3B). Because Myeov2 was present in nucleoplasm but not nucleolus (Fig. 3A and 3D), these data imply that expression of Myeov2 retains L11 in nucleoplasm. To next explore the mechanisms by which Myeov2 controls L11 localization, we examined the interaction between them. Myeov2 clearly bound to L11, and Myeov2ΔFD was also capable of interaction even though its efficiency seems to be weaker than full length (Fig. 3C). Therefore, it is likely that Myeov2 induces suppression of Nedd8 modification through interaction with L11, and segregates L11 from nucleolus. Because L11 is released to nucleoplasm when nucleolus is disrupted following nucleolar stress, we next asked whether expression of Myeov2 induces nucleolar disruption. To examine this, we transfected HeLa cells with EGFP-tagged nucleophosmin (NPM) in the presence or absence of Myeov2 and observed the shape of nucleolus using fluorescence microscope. When nucleolar stress was induced by treatment with 10 nM Actinomycin D (ActD) for 4 hours, EGFP-NPM relocated to nucleoplasm. Myeov2 expression did not induce changes of EGFP-NPM nucleolar localization, indicating that overexpression of Myeov2 *per se* is not sufficient to disrupt nucleolus. However, treatment with 10 nM ActD for 1 hour induced EGFP-NPM relocation in Myeov2-expressing cells, but not control cells (Fig. 3D). Taken together, these data indicate that an increase of Myeov2 expression level does not trigger nucleolar disruption although Myeov2 makes nucleolus vulnerable to stress. Presumably, Myeov2 causes defect in nucleolar integrity by limiting L11 in nucleoplasm, and increases sensitivity to a variety of stresses.

### Expression of Myeov2 Induces an Increase of p53 Sensitivity

**Figure 4:**
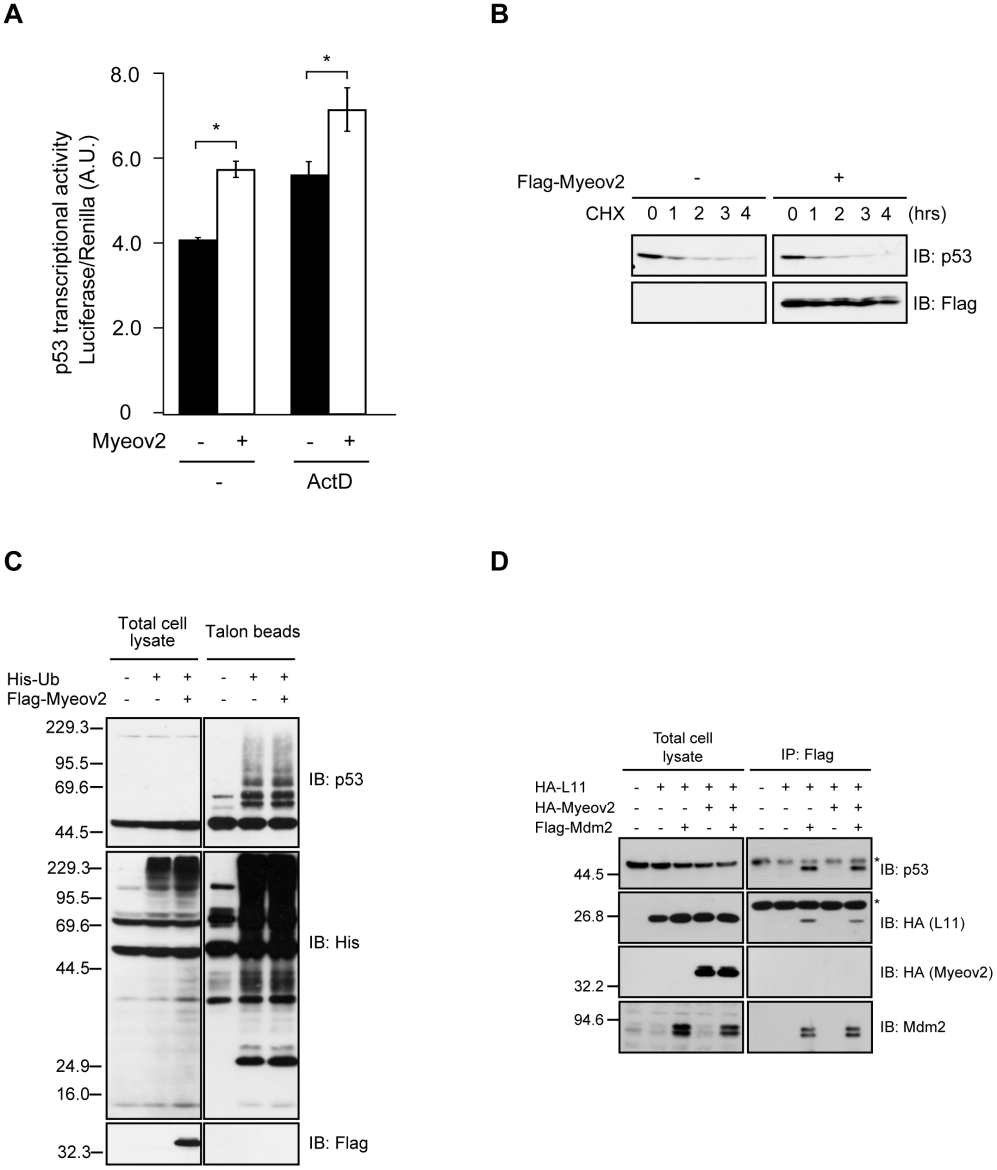
Expression of Myeov2 augments p53 activity independently of ubiquitination. (**A**) HCT116 cells were transfected with either Myeov2 or empty vector, together with p53-promoter-luc plasmids, and were then stimulated with or without 5 nM ActD for 6 hours. p53 promoter activity was analyzed by luciferase assay (n = 3, mean±SEM, * p<0.05 by one-way ANOVA). (**B**) HEK293 cells were transfected with Flag-Myeov2 and were then treated with 10 µM cycloheximide for indicated times. Cell lysates were subjected to immunoblot analyses using anti-p53 and anti-Flag antibodies. (**C**) HEK293T cells were transfected with indicated plasmids. Ubiquitinated proteins were precipitated from denatured cell lysates using Talon metal affinity resin and immunoblotted with anti-His, anti-Flag, and anti-p53 antibodies. (**D**) Co-immunoprecipitation of HA-L11, HA-Myeov2 and endogenous p53 with Flag-Mdm2 in HEK293T cells. Cell lysates were immunoprecipitated with anti-Flag antibody and analyzed using Flag, HA, Mdm2 and p53 antibodies. Asterisks indicate non-specific bands.

It has been reported that L11 stabilizes and activates p53 in response to nucleolar stress [6], [7], [14]. Because p53 is considered to be associated with multiple myeloma [24], [25], and cells become sensitive to nucleolar stress by expression of Myeov2 (Fig. 3D), these observations prompted us to examine whether expression of Myeov2 regulates p53 transcriptional activity. We first introduced Myeov2 plasmids into HCT116 cells and measured p53 transcriptional activity using luciferase assay. Expression of Myeov2 moderately increased p53 activity. In addition, this effect was enhanced after treatment with 5 nM ActD (Fig. 4A). These data are consistent with our notion that Myeov2 induces sensitivity to nucleolar stress. We next investigated whether Myeov2 controls p53 stability. A large amount of p53 disappeared about 1 hour after treatment with 10 µM cycloheximide. The overexpression of Myeov2 did not affect the p53 instability (Fig. 4B). In addition, ubiquitination status of p53 did not change by expression of Myeov2 (Fig. 4C), demonstrating that Myeov2 increases p53 transcriptional activity without affecting its ubiquitination nor stability. To further confirm that Myeov2 does not control p53 ubiquitination, we asked whether Myeov2 is implicated in Mdm2 functions, which is a major ubiquitin ligase of p53. Consistent with our data, expression of Myeov2 did not alter the ubiquitination status of p53 even when Mdm2 was overexpressed (Fig. S4). In addition, Myeov2 did not modulate the interactions of Mdm2 with p53 and L11 (Fig. 4D), supporting the idea that Myeov2 has little influence on the regulation of Mdm2 activity and p53 stability. Taken together, these findings suggest that expression of Myeov2 is not correlated with the inhibition of p53 turnover, but is sufficient to promote p53 activity moderately.

## Discussion

**Figure 5:**
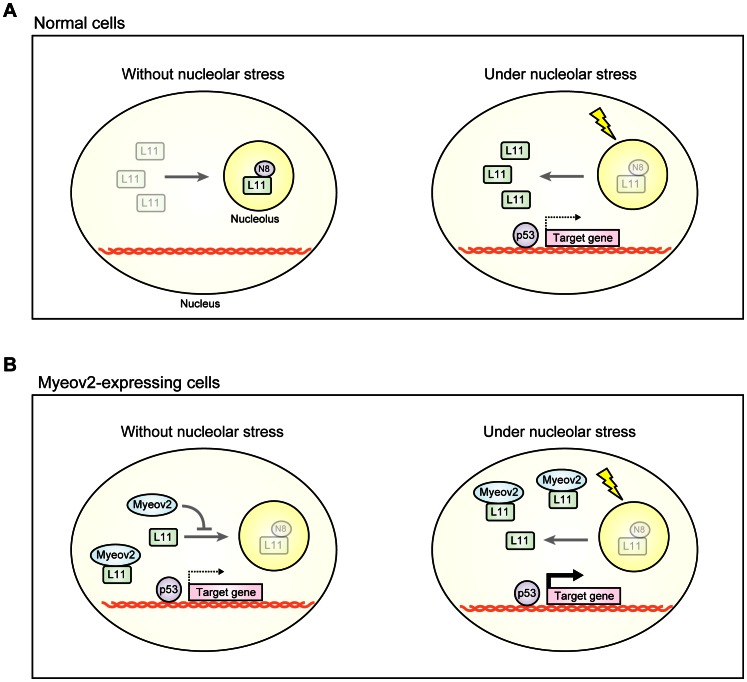
Model for the role of Myeov2 in nucleus. (**A**) Most of L11 is neddylated and localized in nucleolus. In response to nucleolar stress, L11 is deneddylated and relocates to nucleoplasm, leading to activation of p53. (**B**) Myeov2 blocks L11 neddylation by their interaction and suppresses translocation of L11 into nucleolus, and make cells sensitive to stresses.

Nedd8 modification pathway plays pivotal role in a wide variety of cellular systems [32]. Recent studies have reported that nucleolar stress is associated with tumorigenesis through regulation of p53 pathway [1], [2]. However, the mechanisms of how Nedd8 pathway is involved in this machinery remain elusive. Here, we provide several lines of evidence that demonstrate Myeov2 regulates L11 subnucleolar localization that links nucleolar stress to p53 pathway: (1) expression of Myeov2 leads to a global decrease of neddylated proteins; (2) Myeov2 sustains L11 in a lower neddylation status; (3) Knockdown of Myeov2 does not enhance the neddylation level of L11; (4) Myeov2 alters the subnuclear localization of L11; (5) the effect of Myeov2 is independent of Nedd8 deconjugation enzymes, Nedp1 and COP9 signalosome; (6) expression of Myeov2 induces an up-regulation of p53 activity independent of p53 stability. These data demonstrates that Myeov2 is not necessary to inhibit the basal level of L11 neddylation. However, when expression level is increased under abnormal condition, Myeov2 suppresses L11 neddylation through its interaction and segregates L11, allowing an increase of stress sensitivity (Fig. 5). Thus, our findings provide a clue that links Nedd8 pathway to pathogenesis of multiple myeloma.

Nedd8 is a ubiquitin-like protein that conjugates to target proteins and modulates its protein-protein interaction, subcellular localization and enzymatic activity [33], [34]. Since Nedd8 modification is significantly involved in cellular proliferation, it is feasible that abnormality in this pathway is associated with tumorigenesis [32]. In fact, a chemical inhibitor MLN4924 that suppresses Nedd8 E1 enzyme APPBP1/Uba3 inhibits cell cycle progression [35]. Interestingly, MLN4924 is expected to be a potent therapeutic agent for multiple myeloma and clinical trial is on going. The mechanisms of how MLN4924 is effective for multiple myeloma has not been understood, but our data provide a tantalizing prospect that MLN4924 blocks neddylation of ribosomal protein such as L11, allowing vulnerability of nucleolus and activation of p53 pathway.

p53 is one of well-characterized tumor suppressor proteins, which is normally ubiquitinated by Mdm2. So far, it has been reported that several ribosomal proteins are released from nucleolus in response to nucleolar stress and suppress Mdm2 activity, followed by activation of p53 pathway. Although we found that overexpression of Myeov2 retains L11 in nucleoplasm and increases p53 transcriptional activity, the rate of increase in transcriptional activity appears not to be forceful. Because we found that Myeov2 is not sufficient to induce nucleolar disruption, it is possible that the release of other ribosomal proteins caused by nucleolar stress is required for substantial activation of p53. This idea is consistent with our data that Myeov2 did not affect stabilization or ubiquitination of p53 (Fig. 4C). However, precise mechanisms by which Myeov2 augments p53 pathway through regulation of L11 localization remain an issue for future studies.

Myeov2 led to a global decrease of neddylated proteins including ribosomal protein, L11. Of special note is that we did not observe the enhancement of deneddylating activity of COP9 although Myeov2 associates with COP9 signalosome. COP9 signalosome was originally identified as a negative regulator that control photomorphogenesis in plant [36]. Genetic analysis has revealed that mutations in CSN genes exhibit seeding lethal and abnormal light response, demonstrating that COP9 signalosome is essential for a viability of plants [37]. Intriguingly, plant Myeov2 homolog, Smap1 and Smap2, have genetic and physical interactions with a subset of COP9 subunits. An ectopic expression of Smap1 partially compensated the morphological defect of CSN5a mutant in plants, although Smap1 does not deconjugate Nedd8 from Cul1 [38], [39], [40]. This observation is consistent with our data that expression of Myeov2 did not enhance the activity of COP9 signalosome, despite of its interaction (Fig. 1). Meanwhile, COP9 signalosome is associated with embryogenesis, stress response, cellular proliferation and differentiation in mammals [41], [42], [43]. In particular, COP9 signalosome is linked to cell cycle regulation and tumorigenesis. For instance, COP9 subunits such as CSN5 and CSN6 are excessively expressed in cancer cells such as myeloma, leukemia and glioblastoma, and CSN5 is associated with tumor suppressor genes such as p16, p27, p53 and Smad7 [42], [43], [44]. Furthermore, myeloid leukemia factor 1 (MLF1), a novel binding partner of CSN3 that attenuates the ubiquitin ligase activity of COP1, is capable of ubiquitinating p53 [45]. Of note is that chromosomal translocation produces a fusion protein of MLF1 with NPM, and is significantly associated with myelodysplastic syndoromes (MDS), followed by myeloid leukemia [46]. We also found that Myeov2 interacts with COP9 signalosome and activates p53. Since Myeov2 make nucleolus vulnerable to stress stimulation, one potential hypothesis is that Myeov2-COP9 signalosome complex is required for maintenance of nucleolus or p53 activity independently of deneddylation activity. However, understanding the link between Myeov2 and COP9 signalosome will be an important challenge for future experiments.

In addition to a set of CSN subfamily, we also identified other intriguing proteins that co-precipitated with Myeov2, some of them associated with cancer. It has been demonstrated that elevation of calcium in blood is a typical feature of multiple myeloma, and an abnormal calcium influx from extracellular environment is involved in viability of myeloma cells [47]. In this study, we found that Myeov2 binds to calmodulin and its subfamily, calmodulin-like protein 3 (Fig. 1A). Calmodulin is a well-characterized mediator that connects calcium ion to intracellular signaling pathway. Thus, Myeov2 may regulate intracellular calcium signaling through binding to calmodulin, and determine cell fate such as proliferation and transformation. Two other Myeov2 targets, Elongin-B and Elongin-C are components of CRLs that is composed of Cul2, Elongin-B/Elongn-C complex, and von Hippel-Lindau tumor suppressor protein (pVHL). Mutation in *VHL* gene is associated with Von Hippel-Lindau disease, which exhibits severe malignant tumors. Thus, it is likely that Myeov2 also regulates Cul2-type CRLs and its perturbation may cause several cancers. In fact, pVHL increases p53 transcriptional activity mediated by promotion of the binding between p53 and p300 [48]. We found that Myeov2 promotes p53 activity independent of ubiquitination or stabilization of p53. Therefore, Myeov2 may mediate acetylation or phosphorylation of p53, which lead to p53 transcriptional activity in concert with Cul2-type CRLs.

In summary, we provide strong evidence that a novel multiple myeloma-related protein, Myeov2, regulates Nedd8 modification and nucleolar integrity. These findings reveal an unsuspected function for Myeov2 that regulates L11, leading to regulation of p53 stress response pathway. Therefore, it is possible that an increase of Myeov2 expression level may suppress transformation from plasma cells into plasmacytoma by promotion of p53 sensitivity. Taken together, our study suggests a rationale for investigating Myeov2-dependent Nedd8 modification associated with multiple myeloma.

## Materials and Methods

### Materials and Antibodies

Actinomycin D (SIGMA), Talon metal affinity resin (Clontech), MG132 (Peptide Institute), Cyclohexamide (WAKO), and Hoechst 33342 (Life Technologies) were purchased.

Antibodies to Flag (M2, SIGMA), Myc (9E10 Santa Cruz), HA (A190-108A, Bethyl Lab., Inc), HA (Y-11, Santa Cruz), CSN5 (42, BD Transduction Lab.), Cul1 (19/CUL-1, BD Transduction Lab.), Tubulin (DM1A, SIGMA), His (GE Healthcare), p53 (DO-1, Santa Cruz), and Mdm2 (N-20, Santa Cruz) were used for immunoblot analyses. Antibodies to Myc (9E10, Santa Cruz), HA (3F10, Roche), GFP (ab13970, abcam), and DYKDDDDK-tag (#5407, Cell Signaling Technology) were used for immunocytochemistry and Anti-Flag M2-agarose bead (SIGMA) for immunoprecipitation. Antibody to Cul3 has been described previously [49]. Anti-Nedd8 antiserum was raised by immunizing rabbit with recombinant Nedd8.

### Cell Culture and Transfection

HEK293 [50], HEK293T [51], HeLa [52], HCT116 [52] cells were cultured in Dulbecco’s modified Minimal Essential Medium (WAKO) containing 5–10% fetal bovine serum, penicillin (100 units) and streptomycin (100 mg) (P/S). Cells were transfected with plasmids using Lipofectamine 2000 (Invitrogen), FuGENE (Roche) or Polyethyleneimine (Polyscience, Inc.) according to the manufacturer’s instructions.

### Plasmid Construction

pCAGEN-His-Ub, pCAGEN-His-Nedd8, pCAGEN-Bla-HA-Nedp1, PG13-luc, pcDNA3-Flag-Mdm2 constructs were provided by Y. Gotoh (University of Tokyo, Japan) [53]. pEGFP-NPM1 was provided by J. Yanagisawa (University of Tsukuba, Japan). Myeov2 was amplified from human cDNA (NIH Mammalian Gene Collection, Clone ID 100014446, Invitrogen) and subcloned into the BamHI and XhoI sites of pcDNA3-Flag. L11 was amplified from 293 cells cDNA library and subcloned into the BamHI site of pCS4-Myc and pCS4-HA. Deletion mutants of Myeov2 was amplified from pcDNA3-Flag-Myeov2 and subcloned into the BamHI and XhoI sites of pcDNA3-Flag. The primers used for deletion mutants of Myeov2 were as follows: Myeov2 forward, 5′-AAAAGGATCCACATGTGGCGCGCGCCGGAAGC-3′; and reverse, 5′-AAAAAACTCGAGCTACCTCACATCTATGCATG-3′. Myeov2ΔC forward, 5′-AAAAGGATCCACATGTGGCGCGCGCCGGAAGC-3′; and reverse, 5′-AAAAAACTCGAGCTAATGTCATCATCATCAAAAAGATCTTCAAAA-3′.

Myeov2ΔN forward, 5′-GGATCCACATGGTTCCCGCACTGCAGCGAGGGCAGTCG-3′; and reverse, 5′-AAAAAACTCGAGCTACCTCACATCTATGCATG-3′. Myeov2ΔFD forward, 5′-AAAGGATCCACATGTGGCGCGCGCCGGAAGC; Myeov2ΔFD reverse 5′-ATGGACTTGGCAGCCAGCTCAGGACTGCC-3′.

For the shMyeov2 contructs, the oligonucleotides corresponding to the target sequence for Myeov2 (#1 5′-CACAGGACGCTTAAGAAACA-3′, #2 5′-GGACGCTTAAGAAACAGTTTA) were subcloned into pMXII vector.

### Immunoblot Analysis

Cells were lysed in extraction buffer (0.5% NP-40, 20 mM Tris-HCl (pH 7.5), 150 mM NaCl, 1 mM, EDTA, 1 mM DTT) and centrifuged at 14,000 rpm for 5 minutes. The cleared lysates were separated by SDS-PAGE, transferred to PVDF membrane, probed with primary antibodies, and detected with HRP-conjugated secondary antibodies and chemiluminescence reagent (Amersham ECL Plus Western Blotting Detection Reagents, GE Healthcare).

### Immunoprecipitation

The cell lysates (see immunoblot analysis) was rotated with anti-Flag agarose beads (SIGMA) or anti-HA agarose beads (SIGMA) for 4 hours at 4°C. The immunoprecipitants were washed and subjected to immunoblot analyses with indicated antibodies.

### His-tagged Pull down Assay

Cells were transfected with the indicated constructs and lysed in extraction buffer (6 M guanidinium-HCl, 50 mM sodium phosphate buffer (pH 8.0), 300 mM NaCl and 5 mM imidazole). Cell lysates were sonicated briefly and were then incubated with Talon metal affinity resin (Clontech) for 4 hours at 4 C°. The precipitants were washed by buffer (50 mM sodium phosphate buffer (pH 8.0), 300 mM NaCl and 5 mM imidazole) and were then subjected to immunoblot anaylsis.

### Immunocytochemistry

HeLa cells plated on 15 mm coverslips and grown in 12-well plates were fixed with 4% paraformaldehyde in phosphate buffered saline (PBS) for 10 minutes at room temperature. The coverslips were washed in PBS, blocked with 5% bovine serum albumin (BSA), and 0.4% Triton X-100 in PBS, then incubated with the indicated primary antibodies for one hour at room temperature or overnight at 4°C. Following PBS washes, samples were incubated with secondary antibodies (Alexa Fluor 594 anti-mouse IgG (1∶500), Alexa Fluor 488 anti-rat IgG (1∶500) and Alexa Fluor 488 anti-chicken IgG (1∶500)) for 30 minutes at room temperature in blocking solution. Cells were imaged using a fluorescence microscope (Keyence, BIOREVO BZ-9000). Fluorescence images were analyzed using Image J.

### Luciferase Assay

HCT116 cells were transfected with the PG13-Luc reporter plasmid, which contains the firefly luciferase gene driven by p53 promoter, and a renilla luciferase expression plasmid as an internal control. Cell lysates were subsequently assayed for both firefly and renilla luciferase activities with Dual-Luciferase Reporter Assay System (Promega), and the former activity was normalized on the basis of the latter.

## Supporting Information

Figure S1 **Characterization of anti-Nedd8 antiserum.** (A and B) Immunoprecipitation of neddylated proteins. Neddylated proteins were immunprecipiated from HEK293 cells using anti-Nedd8 antiserum and analyzed with anti-Nedd8 antiserum (A) and anti-Ub antibody (FK2, MBL).
(TIF)

Figure S2 **Knockdown of Myeov2 has little effect on L11 neddylation.** (A) HEK293 cells were transfected with expression vectors encoding Flag-Myeov2 and Myeov2 shRNA. Cell lysates were subjected to immunoblot anayses with antibodies to Flag, GFP and Tubulin. The GFP loading control is expressed from the same plasmid as the shRNA. Asterisk indicates non-specific bands. (B) HEK293T cells were transfected with indicated plasmids. Neddylated proteins were pulled down from denatured cell lysates using Talon metal affinity beads, and analyzed by immunoblot analyses using antibodies to His and Myc.
(TIF)

Figure S3 **Myeov2 does not interact with Nedp1.** (A and B) Co-immunoprecipitation experiment of HA-Nedp1 and Flag-Myeov2. HEK293T cells were co-transfected with HA-Nedp1 and either Flag-Myeov2 or Flag-Myeov2DFD. Cell lysates were immunprecipiated with either anti-HA or anti-Flag antibodies and analyzed using Flag and HA antibodies.
(TIF)

Figure S4 **Myeov2 does not alter Mdm2-mediated p53 ubiquitination.** (A) HEK293T cells were transfected with indicated plasmids. Ubiquitinated proteins were pulled down from denatured cell lysates using Talon metal affinity beads, and analyzed by immunoblot analyses using antibodies to His, HA, p53 and Mdm2.
(TIF)
