# Supplementary figures and images for: Myeloma Overexpressed 2 (Myeov2) Regulates L11 Subnuclear Localization through Nedd8 Modification
Source: PLoS One. 2013 Jun 12;8(6):e65285. doi: 10.1371/journal.pone.0065285 (PMC3680436; doi:10.1371/journal.pone.0065285)

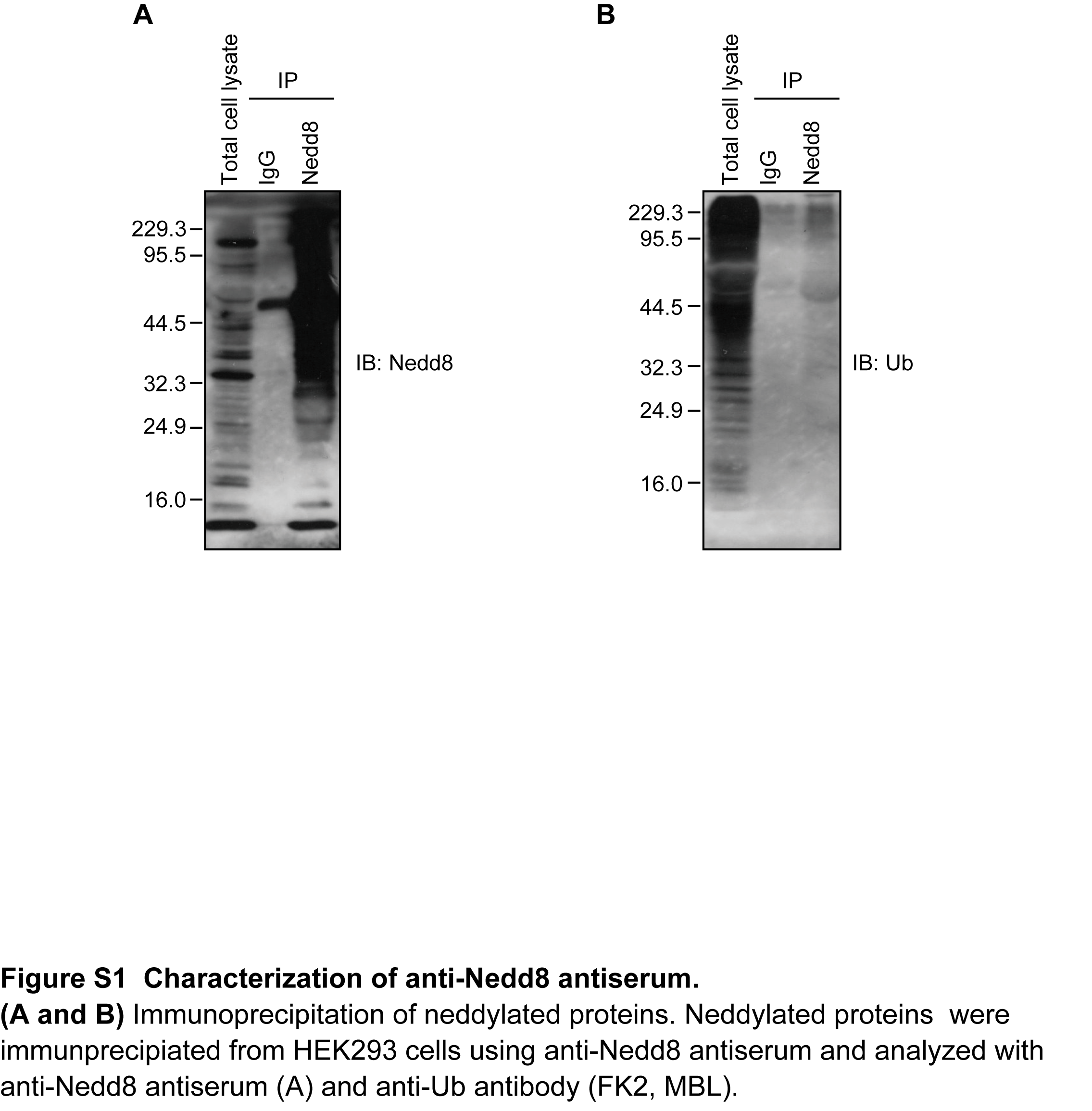

Supplement: Figure S1 — Characterization of anti-Nedd8 antiserum. (A and B) Immunoprecipitation of neddylated proteins. Neddylated proteins were immunprecipiated from HEK293 cells using anti-Nedd8 antiserum and analyzed with anti-Nedd8 antiserum (A) and anti-Ub antibody (FK2, MBL). (TIF) [file pone.0065285.s001.tif]

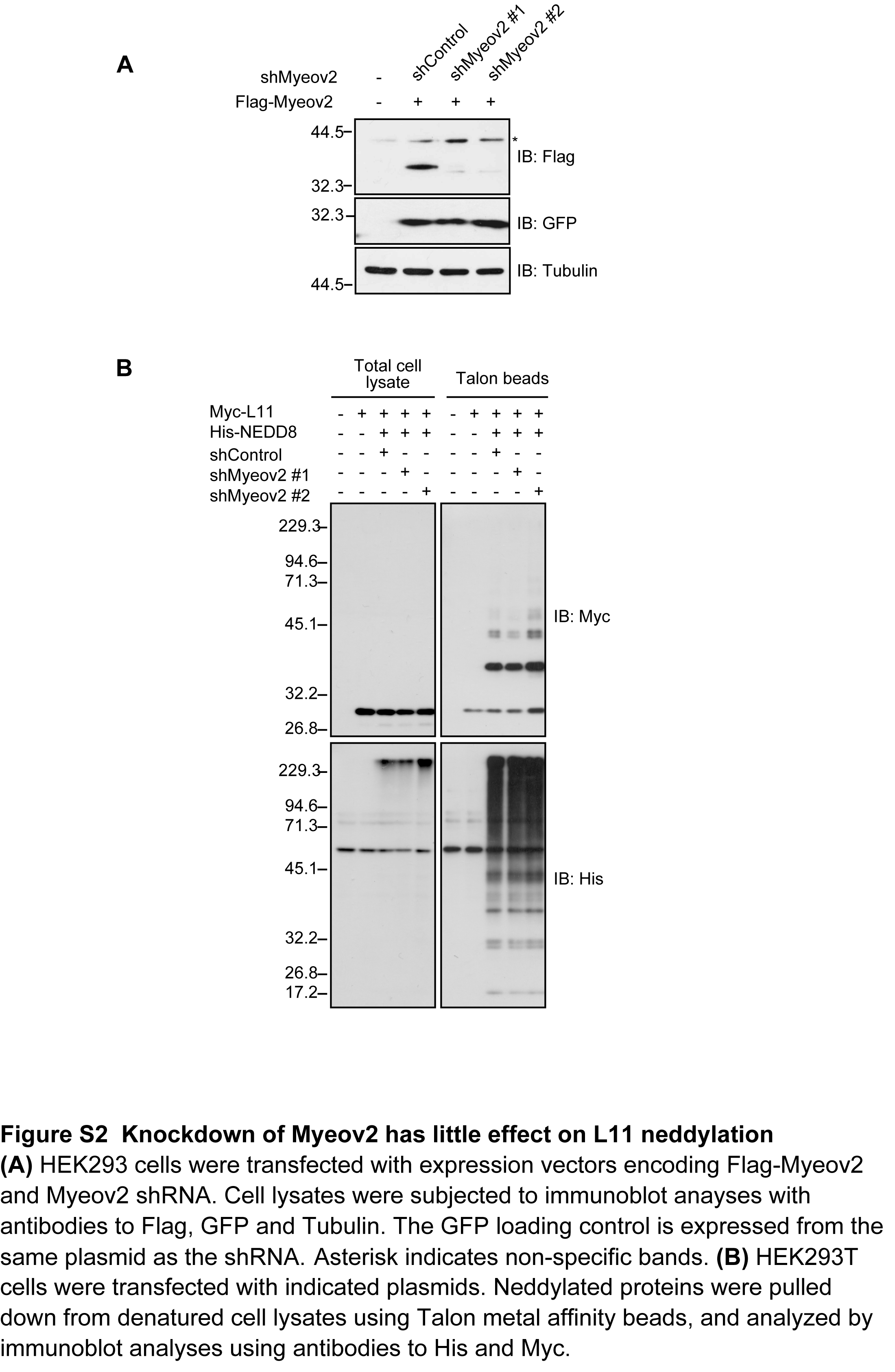

Supplement: Figure S2 — Knockdown of Myeov2 has little effect on L11 neddylation. (A) HEK293 cells were transfected with expression vectors encoding Flag-Myeov2 and Myeov2 shRNA. Cell lysates were subjected to immunoblot anayses with antibodies to Flag, GFP and Tubulin. The GFP loading control is expressed from the same plasmid as the shRNA. Asterisk indicates non-specific bands. (B) HEK293T cells were transfected with indicated plasmids. Neddylated proteins were pulled down from denatured cell lysates using Talon metal affinity beads, and analyzed by immunoblot analyses using antibodies to His and Myc. (TIF) [file pone.0065285.s002.tif]

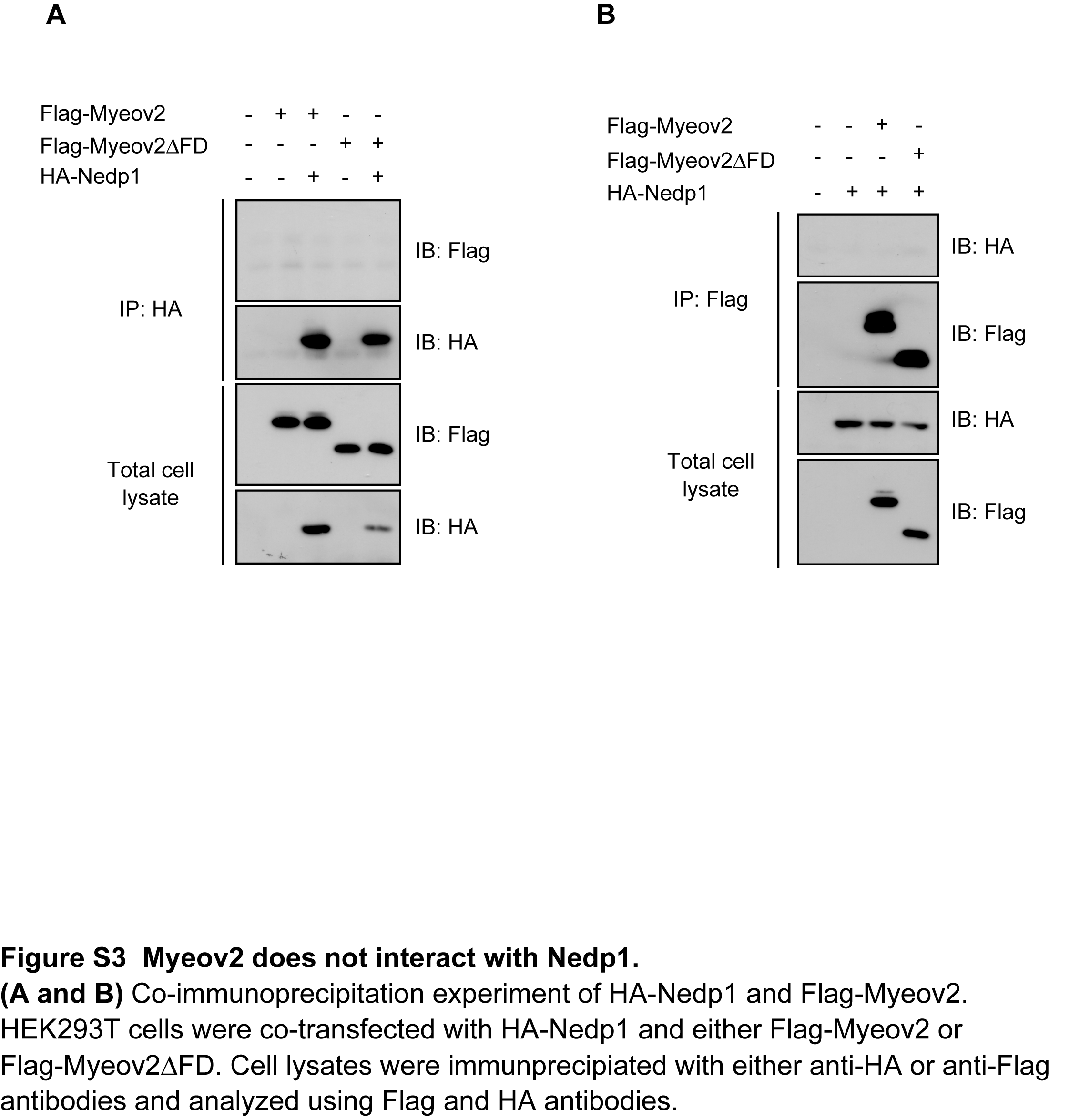

Supplement: Figure S3 — Myeov2 does not interact with Nedp1. (A and B) Co-immunoprecipitation experiment of HA-Nedp1 and Flag-Myeov2. HEK293T cells were co-transfected with HA-Nedp1 and either Flag-Myeov2 or Flag-Myeov2DFD. Cell lysates were immunprecipiated with either anti-HA or anti-Flag antibodies and analyzed using Flag and HA antibodies. (TIF) [file pone.0065285.s003.tif]

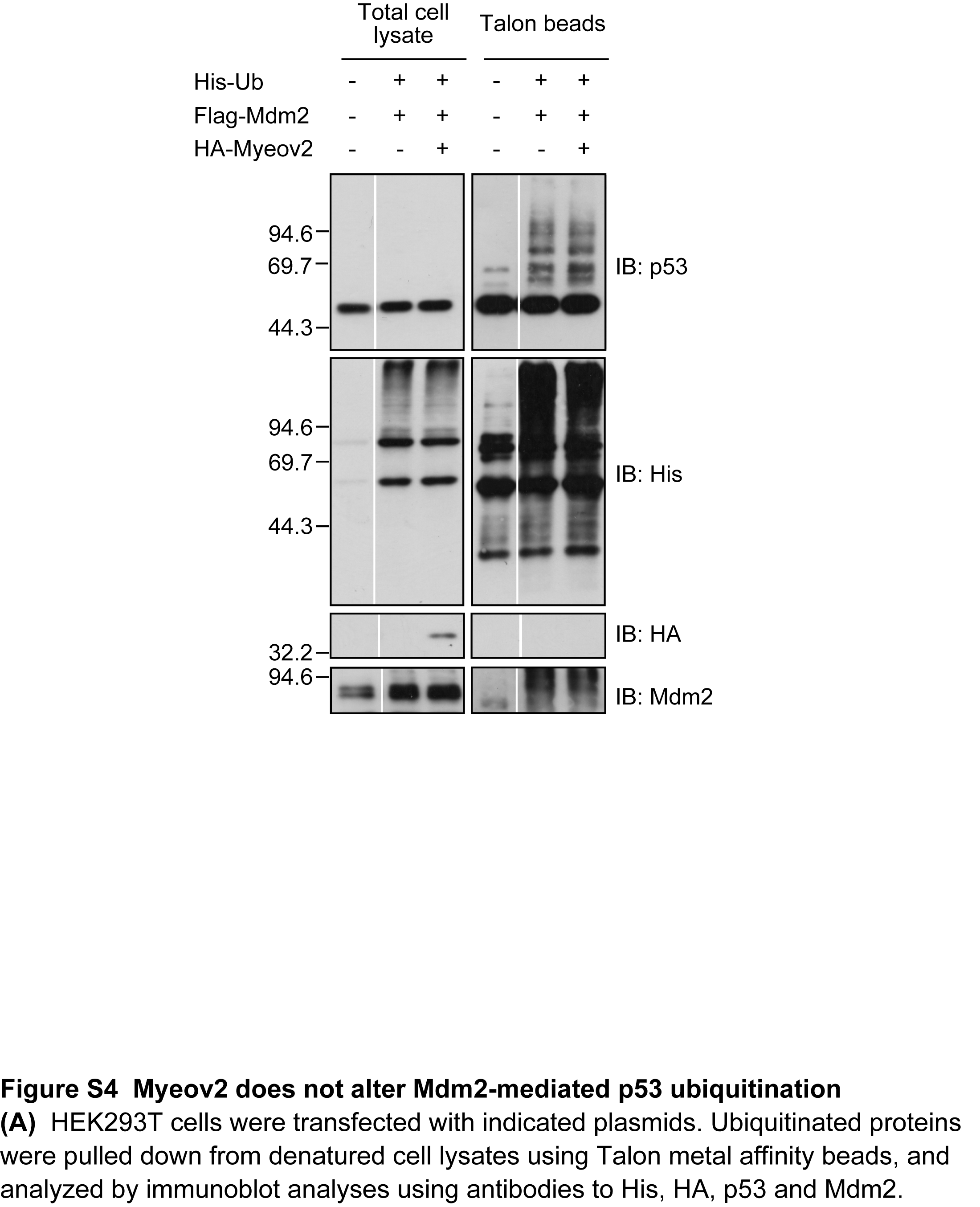

Supplement: Figure S4 — Myeov2 does not alter Mdm2-mediated p53 ubiquitination. (A) HEK293T cells were transfected with indicated plasmids. Ubiquitinated proteins were pulled down from denatured cell lysates using Talon metal affinity beads, and analyzed by immunoblot analyses using antibodies to His, HA, p53 and Mdm2. (TIF) [file pone.0065285.s004.tif]
